# On the Effects of Hot Forging and Hot Rolling on the Microstructural Development and Mechanical Response of a Biocompatible Ti Alloy

**DOI:** 10.3390/ma5081439

**Published:** 2012-08-20

**Authors:** Yoshimitsu Okazaki

**Affiliations:** Advanced Biomaterials Group, Human Technology Research Institute, National Institute of Advanced Industrial Science and Technology, 1-1-1 Higashi, Tsukuba-shi, Ibaraki 305-8566, Japan; E-Mail: y-okazaki@aist.co.jp; Tel.: +81-29-861-7179; Fax: +81-29-861-6149

**Keywords:** titanium alloy, marketing approval, manufacturing equivalency, microstructure, mechanical property, hot forging, continuous hot rolling, fatigue property

## Abstract

Zr, Nb, and Ta as alloying elements for Ti alloys are important for attaining superior corrosion resistance and biocompatibility in the long term. However, note that the addition of excess Nb and Ta to Ti alloys leads to higher manufacturing cost. To develop low-cost manufacturing processes, the effects of hot-forging and continuous-hot-rolling conditions on the microstructure, mechanical properties, hot forgeability, and fatigue strength of Ti-15Zr-4Nb-4Ta alloy were investigated. The temperature dependences with a temperature difference (ΔT) from β-transus temperature (Tβ) for the volume fraction of the α- and β-phases were almost the same for both Ti-15Zr-4Nb-4Ta and Ti-6Al-4V alloys. In the α-β-forged Ti-15Zr-4Nb-4Ta alloy, a fine granular α-phase structure containing a fine granular β-phase at grain boundaries of an equiaxed α-phase was observed. The Ti-15Zr-4Nb-4Ta alloy billet forged at Tβ-(30 to 50) °C exhibited high strength and excellent ductility. The effects of forging ratio on mechanical strength and ductility were small at a forging ratio of more than 3. The maximum strength (σ_max_) markedly increased with decreasing testing temperature below Tβ. The reduction in area (R.A.) value slowly decreased with decreasing testing temperature below Tβ. The temperature dependences of σ_max_ for the Ti-15Zr-4Nb-4Ta and Ti-6Al-4V alloys show the same tendency and might be caused by the temperature difference (ΔT) from Tβ. It was clarified that Ti-15Zr-4Nb-4Ta alloy could be manufactured using the same manufacturing process as for previously approved Ti-6Al-4V alloy, taking into account the difference (ΔT) between Tβ and heat treatment temperature. Also, the manufacturing equivalency of Ti-15Zr-4Nb-4Ta alloy to obtain marketing approval of implants was established. Thus, it was concluded that continuous hot rolling is useful for manufacturing α-β-type Ti alloy.

## 1. Introduction

Orthopaedic implants require biomechanical and biochemical compatibilities as well as biological safety. Therefore, many types of metallic orthopaedic device, which are manufactured from metallic materials with excellent mechanical property and structural stability, are used worldwide in orthopaedics. Titanium (Ti) materials have been widely used to replace failed hard tissues, namely, bone screws, bone plates, compression hip screws, intramedullary fixations, short femoral nails, artificial hip joints, artificial knee joints, spinal instruments, and dental implants. Recent studies of Ti alloys have been carried out, assuming their long-term use in the human body and possible long-term health problems associated with the release of toxic ions and fatigue fracture [1,2,3,4,5,6]. The relationship between the cytocompatibility and polarization resistance of various pure metals has previously been summarized [7,8,9,10]. The cytotoxicity of V ions is observed from a medium concentration of 0.1 mass ppm. Among the 70 metals in the periodic table, only Ti and Zr show excellent cytocompatibility to both soft-tissue-derived mouse fibroblast cells and bone-derived mouse osteoblast cells [8]. Adding a small amount of alternative metal elements improves the quality of Ti alloys. However, excess addition of Nb and Ta to Ti alloys may increase manufacturing cost. Ti alloys are categorized into three types according to microstructure: α (alpha)-type alloy having a hexagonal close-packed structure, hcp; β (beta)-type having a body-centred cubic structure, bcc; and α-β-type having a mixed structure of the α- and β-phases. Among the α-β-type Ti alloys, Ti-6Al-4V alloy has been extensively used for various orthopaedic applications. The α-β-type Ti alloy demonstrates better fatigue characteristics than the β-type alloy. As for another α-β-type Ti alloy, the Ti-15Zr-4Nb-4Ta alloy has been developed in Japan as a highly biocompatible alloy for long-term biomedical applications [11,12,13,14,15,16,17,18,19,20,21,22] and is now standardized in JIS T 7401-4 [23]. Medical devices (implants) with a new material is should show proof that its manufacturing process is equivalent to that of previously approved implants (e.g., Ti-6Al-4V) for marketing approval of implants specified in the Pharmaceutical Affairs Law. In this study, to prove manufacturing equivalency of Ti-15Zr-4Nb-4Ta alloy to approved manufacturing processes for Ti-6Al-4V alloy, and to develop advanced manufacturing processes such as die forging, the mechanical property and deformation capability from room temperature up to high temperatures as well as the effects of hot forging and continuous hot-rolling conditions on the microstructure, mechanical properties and fatigue strength of Ti-15Zr-4Nb-4Ta alloy were investigated. These properties were compared with those of Ti-6Al-4V alloy.

## 2. Materials and Methods

### 2.1. Alloy Specimens

**Figure 1:**
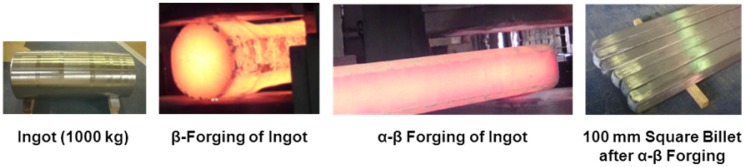
Ti-15Zr-4Nb-4Ta alloy B ingot and 100 mm square billets after β and α-β forgings.

Vacuum-arc melting was performed on the Ti-15Zr-4Nb-4Ta alloy (JIS T 7401-4) for medical implants [23]. A Ti-15Zr-4Nb-4Ta alloy ingot is homogenized at approximately 1200 °C for more than 6 h, and β-forged at the same temperature to a forging ratio (cross section before forging/cross section after forging) of more than 3. Secondly, β-forging, while controlling the grain growth of the β-phase, is conducted to make the β-phase as small as possible at 1050 to 1100 °C, in proportion to the size of the billet and forging ratio. Afterwards, α-β-forging at Tβ-(35 to 50) °C is conducted to obtain the α- and β-structures, by decoupling the fine β-phase. Tβ indicates the β-transus temperature (100 vol% β phase). 100 mm square and 30 mm square billets were manufactured by α-β-forging. The specific forging was performed under atmospheric conditions using a 1200-ton forging machine. To prevent the edge of the billet from cracking caused by heat loss, forging time was minimized by adjusting forging reduction and forging width/speed. The reheating of ingot and forging were repeated once or twice to optimize ingot size and microstructure. In between β- and α-β-forgings, the billet surface was ground with a grinder to prevent cracking due to the oxide scale formed on the ingot surface. Figure 1 shows the Ti-15Zr-4Nb-4Ta alloy ingot and α-β-forged billet (100 mm square). Table 1 shows the chemical compositions and Tβ values of the Ti-15Zr-4Nb-4Ta alloy billets used in this study. Two types (A and B) of Ti-15Zr-4Nb-4Ta alloy with different chemical compositions of trace elements (O and N) were melted and hot-forged.

**Table 1:** Chemical compositions (mass%) and Tβ values of Ti-15Zr-4Nb-4Ta alloys used.

Alloy	Zr	Nb	Ta	Pd	Fe	O	N	H	C	Ti	Tβ (°C)
A	15.52	4.0	4.0	0.18	0.026	0.200	0.042	0.0011	<0.005	Bal.	810 °C
B	16.55	4.0	3.9	0.01	0.040	0.275	0.085	0.0010	0.007	Bal.	830 °C

### 2.2. Solution Treatment and Microstructural Observation

The test specimens for solution treatment were prepared from α-β-forged billets (forged at 780 °C, forging ratio of more than 3) of Ti-15Zr-4Nb-4Ta alloys A and B. The test specimens were solution-treated at 845, 840, 835, 830, 825, 820, 815,810, 805, 800, 795, 780, 760, 740, 720, 700, 650, 600 and 500 °C for 1 h, and then quenched in water. The solution-treated samples were embedded in resin and polished to a mirrorlike finish with 200 to 4200 grit waterproof emery paper and OP-S suspension. Then, each sample was etched with nitric acid solution containing 10 vol% H_2_O_2_ and 3 vol% hydrogen fluoride for microstructural observation. The microstructures of Ti-15Zr-4Nb-4Ta alloys was analyzed by optical microscopy, scanning electron microscopy (SEM) and transmission electron microscopy (TEM). Microstructural observation was carried out to investigate the changes in α (hcp crystal structure)-and β (bcc crystal structure)-matrix structures. TEM was performed using disc specimens of 3 mm diameter, which were prepared by electrolytic polishing with 95% methanol+5% perchloric acid solution. Also, three SEM images were taken of Ti-15Zr-4Nb-4Ta alloys A and B after etching for image analysis. The microstructures of the solution-treated Ti-15Zr-4Nb-4Ta alloys A and B were traced with transparency, and all the phases except the α-phase were blackened out for image analysis. The volume fractions of the α- and β-phases, the grain size of the α-phase and the distribution (number) of the aspect ratio (length of short axis/length of long axis) of the α-phase were measured by image analysis with three traced SEM images.

### 2.3. Hot-Forging Test

To examine the optimal α-β-forging conditions for Ti-15Zr-4Nb-4Ta alloy, an α-β-forging test was conducted with Ti-15Zr-4Nb-4Ta alloy A. 100 mm square billets were β-annealed at 1000 °C for 1 h, and then air-cooled. Each billet was hot-forged at forging ratios of 2, 3, 5, 10 and 20 at starting temperatures of 800 (Tβ-10), 780 (Tβ-30), 760 (Tβ-50), 740 (Tβ-70) and 720 (Tβ-90) °C, respectively. After the α-β forging, all the Ti billets were annealed at 650 °C for 2 h. To investigate the effects of forging ratio and forging temperature on mechanical properties, a room-temperature tensile test was conducted with each forged Ti-15Zr-4Nb-4Ta alloy A. The microstructures of the α-β-forged Ti-15Zr-4Nb-4Ta alloy were analyzed by optical microscopy, SEM and TEM. The volume fractions of the α- and β-phases, the grain size of the α-phase and the distribution of the aspect ratio of the α-phase were measured by image analysis with three traced SEM images.

### 2.4. Hot-Rod-Rolling Test

**Figure 2:**
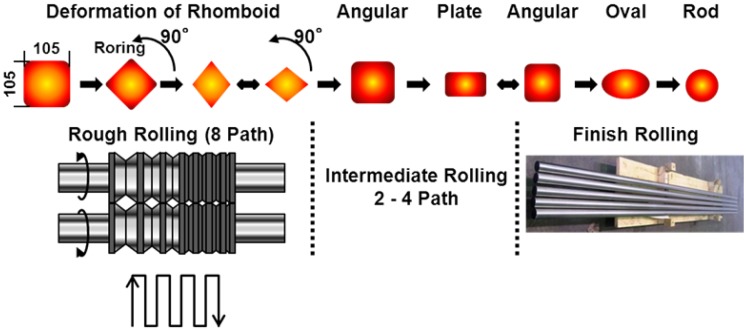
Schematic illustration of continuous hot rolling of Ti alloy to rod specimen.

To develop low-cost manufacturing processes for Ti-15Zr-4Nb-4Ta alloy, a continuous hot-rolling test was conducted with 105 mm square × 1-m-long billets of Ti-15Zr-4Nb-4Ta alloy B. Figure 2 shows a schematic illustration of a continuous hot-rolling process for rod billets. After maintaining them at Tβ-50 °C (780 °C) for 2 h, the billets were hot-rolled continuously in the α-β temperature region (below Tβ) at a low rolling speed to prevent an increase in the internal temperature of the rolling rod. The rod diameters after continuous hot rolling were 50, 35, 30, 18 and 12 mm. After α-β rolling, all the hot-rolled Ti rods were annealed at 700 °C for 2 h. To investigate the effects of hot rolling on mechanical properties, room-temperature tensile test and microstructural observation were conducted with each hot-rolled Ti-15Zr-4Nb-4Ta alloy rod in accordance with standard procedures for implantable metal.

### 2.5. Mechanical Tests

**Figure 3:**
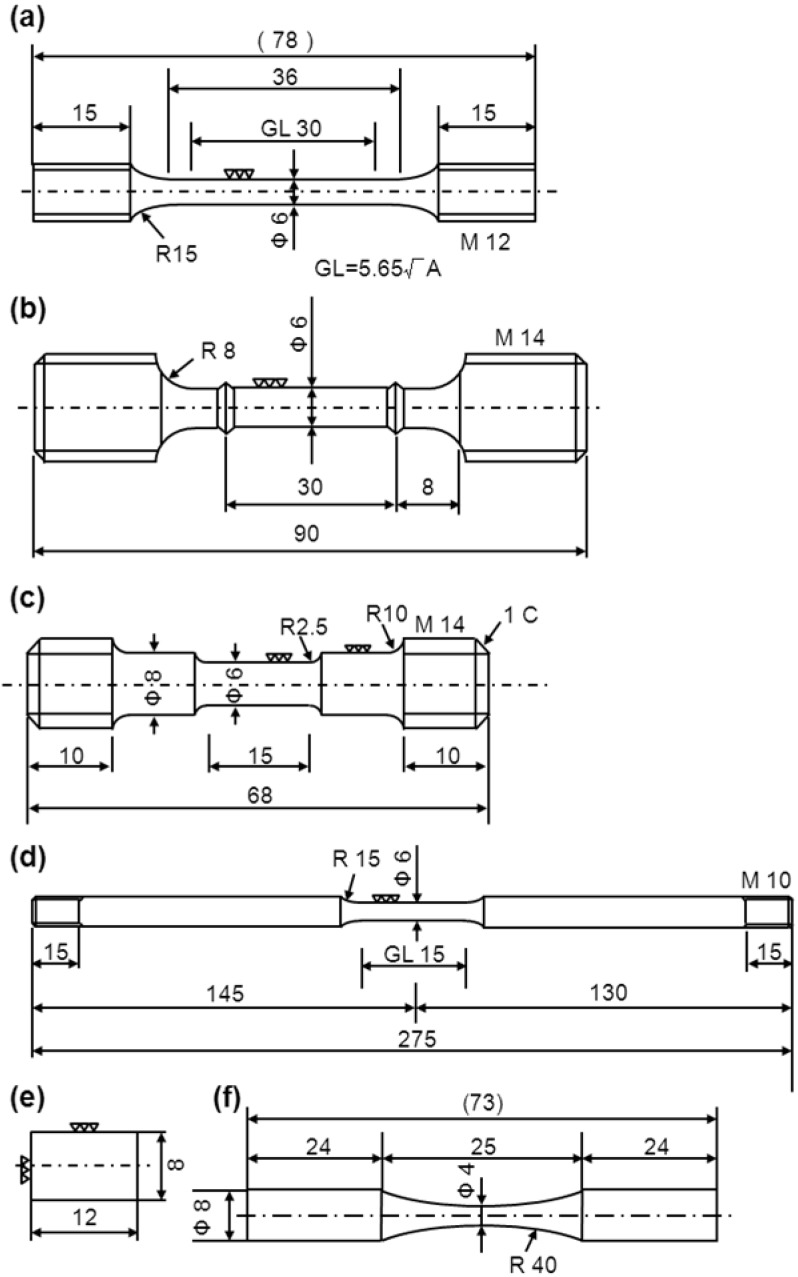
Dimensions of specimens for various mechanical tests. (**a**) Room-temperature tensile test (uniform rod); (**b**) high-temperature tensile test (100–500 °C); (**c**) high-temperature tensile test (600–900 °C) and tensile Gleeble test (550–1250 °C); (**d**) high-temperature tensile test (950–1100 °C); (**e**) compressive Gleeble test (100–1200 °C); (**f**) hourglass-shaped-rod fatigue test.

To estimate the mechanical properties and hot forgeability of Ti-15Zr-4Nb-4Ta alloy, various tensile and compressive tests were conducted within a temperature range from room temperature up to a high temperature of 1250 °C. The room-temperature tensile test, high-temperature tensile test, tensile Gleeble test and compressive Gleeble test were performed with Ti-15Zr-4Nb-4Ta alloy. Moreover, the effects of strain rate and anisotropy caused by forging on mechanical properties at room temperature were also examined. Figure 3 shows the dimensions of the specimens for various mechanical tests. Three specimens each for tensile test at room temperature were cut from the center of each billet with their longitudinal direction (L-direction) parallel to the forging or rolling direction. Also, to examine the effect of anisotropy on room-temperature mechanical properties, two small specimens of 1.5 mm diameter and 9 mm gauge length each for the tensile test were cut from their perpendicular direction (T-direction) parallel to the forged direction as well as cut from their L-direction.

In the room-temperature tensile test, normal specimens of 6 mm diameter and 30 mm gauge length were mainly pulled at a crosshead speed of 0.15 mm/min (0.5%/min) until a proof strength of 0.2% was reached. Crosshead speed was then changed and maintained at 180 mm/min (0.1 s^−1^) until the specimen fractured. The effect of strain rate on mechanical properties was examined with two specimens at room temperature. Firstly, to investigate the effect of strain rate on 0.2% proof strength, specimens of 6 mm diameter and 30 mm gauge length were pulled at crosshead speeds of 0.5, 1.25, 2.4 and 5.6%/min until a proof strength of 0.2% was reached. Then, crosshead speed was maintained at 10%/min until the specimen fractured. Secondly, to investigate the effect of strain rate on ultimate tensile strength, the tensile specimens were pulled at a crosshead speed of 0.5%/min until a proof strength of 0.2% was reached. Crosshead speed was then changed and maintained at 1.25, 2.4, 5.6 and 10%/min until the specimen fractured. Each room- temperature tensile test was performed with three or two specimens. 0.2% proof strength (σ_0.2%PS_), ultimate tensile strength (σ_UTS_), total elongation (T.E.) and reduction in area (R.A.) were measured using conventional stress (MPa)-conventional strain (%) curves. Mean and standard deviation were calculated with the results of three or two specimens.

The specimens for the high-temperature tensile and Gleeble tests were cut from the center of rod specimens with their L-direction parallel to the rolling direction of the Ti-15Zr-4Nb-4Ta alloy B rod (18 mm diameter). In high-temperature tensile tests at 100, 200, 300, 400 and 500 °C in atmosphere, specimens of 6 mm diameter and 30 mm gauge length were pulled at a crosshead speed of 0.15 mm/min (0.5%/min) until a proof strength of 0.2% was reached. Crosshead speed was then changed to 180 mm/min (0.1 s^−1^) and maintained until the specimen fractured. For the high-temperature tensile tests at 600, 700, 750, 800, 850, 900, 950, 1000, 1050 and 1100 °C in Ar atmosphere, specimens of 6 mm diameter and 15 mm gauge length were pulled at a crosshead speed of 90 mm/min (0.1 s^−1^) until the specimen fractured. Moreover, for the high-temperature Gleeble tensile tests at 550, 600, 650, 700, 750, 800, 850, 900, 950, 1000, 1050, 1100, 1150, 1200 and 1250 °C in Ar atmosphere, specimens of 6 mm diameter and 15 mm gauge length were pulled at a crosshead speed of 90 mm/min (0.1 s^−1^) until the specimen fractured. In the high-temperature tensile test, σ_0.2%PS_, σ_UTS_, T.E. and R.A. were measured with conventional stress-conventional strain curves and conventional stress (MPa)- or applied load (N)-crosshead displacement (mm) curves. However, 0.2% proof strength was measured below 500 °C. On the other hand, in the tensile Gleeble tests, maximum tensile strength (σ_max_) and R.A. were measured using applied load (kN)-crosshead displacement (mm) curves. Mean and standard deviation were calculated with the results of two specimens.

In the high-temperature compressive Gleeble tests at 100, 200, 300, 400, 500, 600, 700, 800, 900, 1000, 1100 and 1200 °C, specimens of 8 mm diameter and 12 mm length were compressed at a strain rate of 0.1 or 1 s^−1^ in Ar atmosphere. Each Gleeble test was performed with two specimens. Maximum compressive strength (σ_max_) and compressive strain (%) were measured using compressive applied load (kN)-crosshead displacement (mm) curves. Mean and standard deviation were calculated with the results of two specimens.

### 2.6. Fatigue Test

To investigate the effect of forging conditions on fatigue strength, fatigue tests were conducted. Figure 3f shows hourglass-shaped rod specimens cut from each annealed Ti-15Zr-4Nb-4Ta alloy A at 650 °C for 2 h after α-β forging at various forging temperatures at forging ratios of more than 3. The specimens were machined with their longitudinal direction parallel to the forging direction. To remove the inner strain formed on the surfaces of the specimens during the manufacturing process, the specimen surfaces were fully ground using 600 and 1200 grit waterproof emery papers in the direction parallel to the test specimen.

An electrohydraulic-servo testing machine was used. The fatigue tests were carried out in the tension-to-tension mode with a sine wave in air atmosphere. The testing conditions were as follows: a stress ratio (R = [minimum stress)/(maximum stress)] of 0.1 and a frequency of 1 Hz. To obtain the profiles of maximum stress (maximum applied load/area of cross section) *vs*. the number of cycles (S-N curves), the specimens were cycled at a constant load amplitude until failure or up to 10^7^ cycles when no failure occurred.

## 3. Results and Discussion

### 3.1. Effect of Solution-Treatment on Microstructure

**Figure 4:**
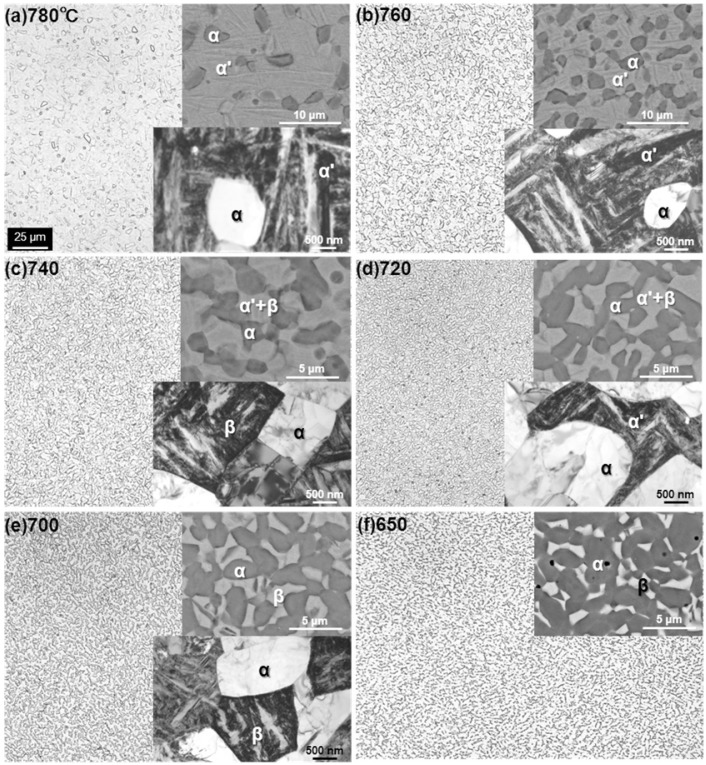
Comparison of optical microscopy, scanning electron microscopy (SEM) and transmission electron microscopy (TEM) images of Ti-15Zr-4Nb-4Ta alloy solution-treated at (**a**) 780; (**b**) 760; (**c**) 740; (**d**) 720; (**e**) 700; (**f**) and 650 °C for 1 h, and then quenched in water.

**Figure 5:**
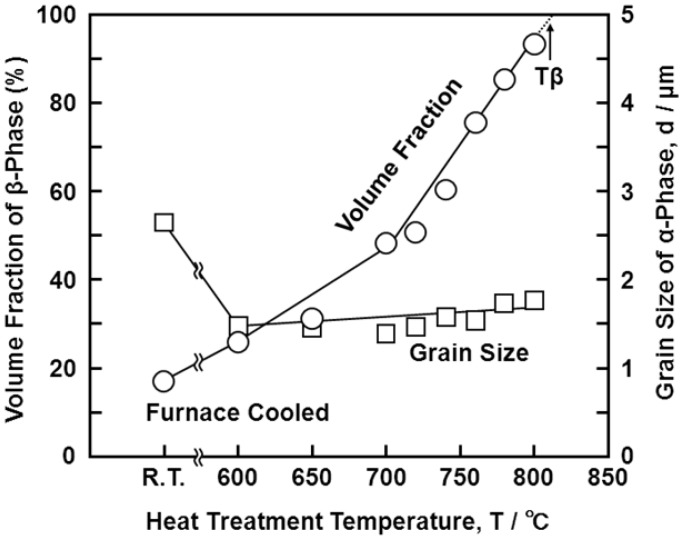
Effects of solution treatment temperature on volume fraction of β-phase and grain size of α-phase of Ti-15Zr-4Nb-4Ta alloy (A).

**Figure 6:**
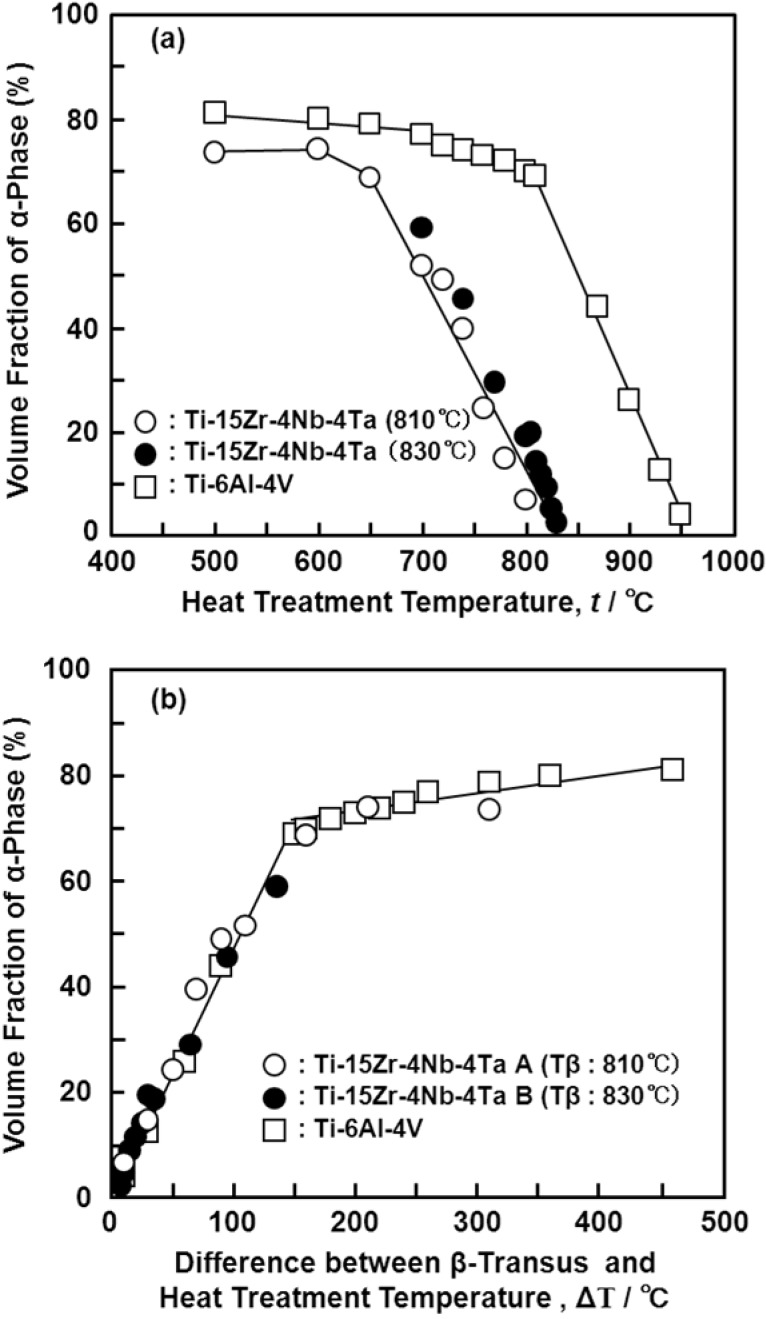
(**a**) in volume fraction of α-phase as a function of heat treatment temperature; and (**b**) difference (ΔT) between Tβ and heat treatment temperature.

Figure 4 shows optical micrographs, and scanning electron microscopy (SEM) and transmission electron microscopy (TEM) images of the Ti-15Zr-4Nb-4Ta alloy A after solution treatment at intervals of 20 °C from 650 up to 780 °C for 1 h, followed by water cooling. The microstructure of the Ti-15Zr-4Nb-4Ta alloy solution-treated below Tβ consisted of an α’-martensite (hcp) phase containing a primary α (hcp)-phase. This α’-martensite was caused by martensitic transformation from the bcc phase to the hcp phase by water cooling. The α-phase increased with decreasing solution treatment temperature. On the other hand, the β-phase, which is stable at high temperatures, decreased with decreasing solution treatment temperature. This β**-**phase appears white in the SEM images shown in Figure 4e,f. TEM observation and electron beam diffraction analysis confirmed the β-phase at grain boundaries of the α-phase, as shown in Figure 4e. A similar α-β structure can be seen in the TEM image of the Ti-6Al-4V alloy annealed at 700 °C for 2 h [24]. The annealed Ti-6Al-4V alloy consisted of an α-phase matrix with approximately 20 vol% β-phase. Figure 5 shows the effects of solution treatment temperature on the volume fraction of the β-phase and the grain size of the α-phase. The volume fraction of the β-phase is close to 100 vol% at a heat treatment temperature of 810 °C. The grain size of the α-phase is approximately 1.5 μm, and the effect of the heat treatment temperature on the grain size of the α-phase is small. Figure 6 shows the changes in the volume fraction of the α-phase with each heat treatment temperature [Figure 6a] and with the temperature difference (ΔT) between Tβ and each heat-treatment temperature [Figure 6b]. The results of the Ti-6Al-4V alloy shown in Figure 6 are cited from the literature [25]. The Tβ of the Ti-15Zr-4Nb-4Ta alloy is much lower than that of the Ti-6Al-4V alloy. The recorded changes in the volume fraction of the α-phase for both Ti-15Zr-4Nb-4Ta and Ti-6Al-4V alloys were almost the same, as shown in Figure 6b. Thus, Tβ is an important parameter for understanding the microstructural change of (α-β)-type Ti alloy, and the microstructure at each temperature can be predicted using the temperature difference (ΔT) from Tβ. β-transus can be predicted using the following formula and the chemical composition of each element (percent by mass) [9]:

Tβ/°C = 848 − 4.2[%Zr] − 5.5[%Ta] − 6.3[%Nb] − 76[%Pd] + 343[%O] + 600[%N]
(1)

### 3.2. Effect of Hot Forging on Microstructure

**Figure 7:**
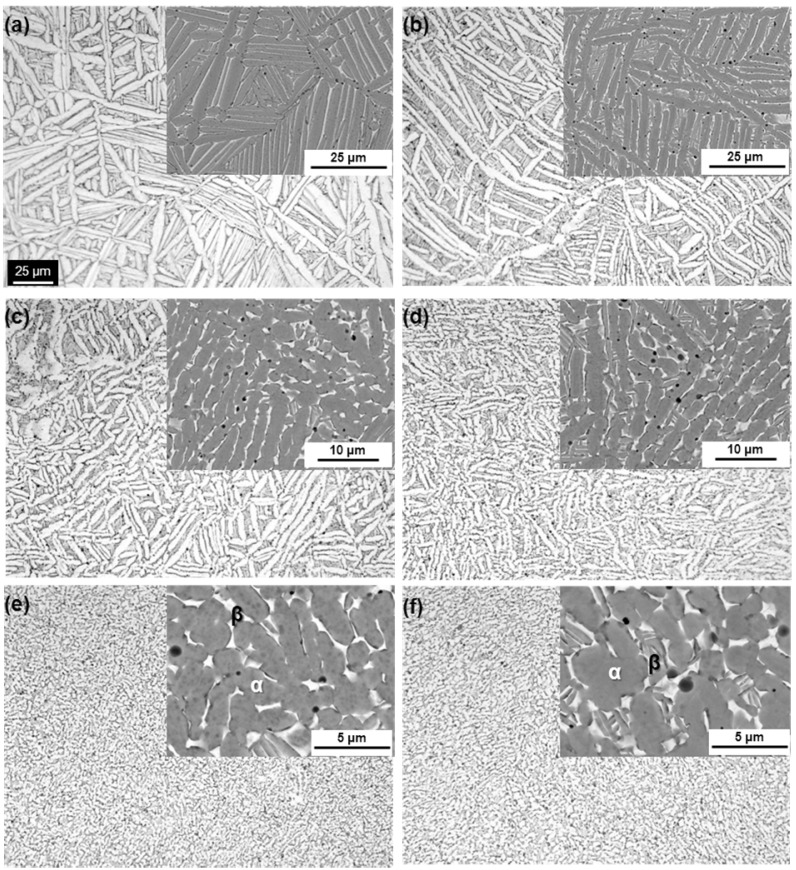
Comparison of optical microscopy and SEM images of Ti-15Zr-4Nb-4Ta alloy A forged at 760 °C. (**a**) β-Annealed; (**b**) forging ratio of 2; (**c**) forging ratio of 3; (**d**) forging ratio of 5; (**e**) forging ratio of 10 and (**f**) forging ratio of 20.

**Figure 8:**
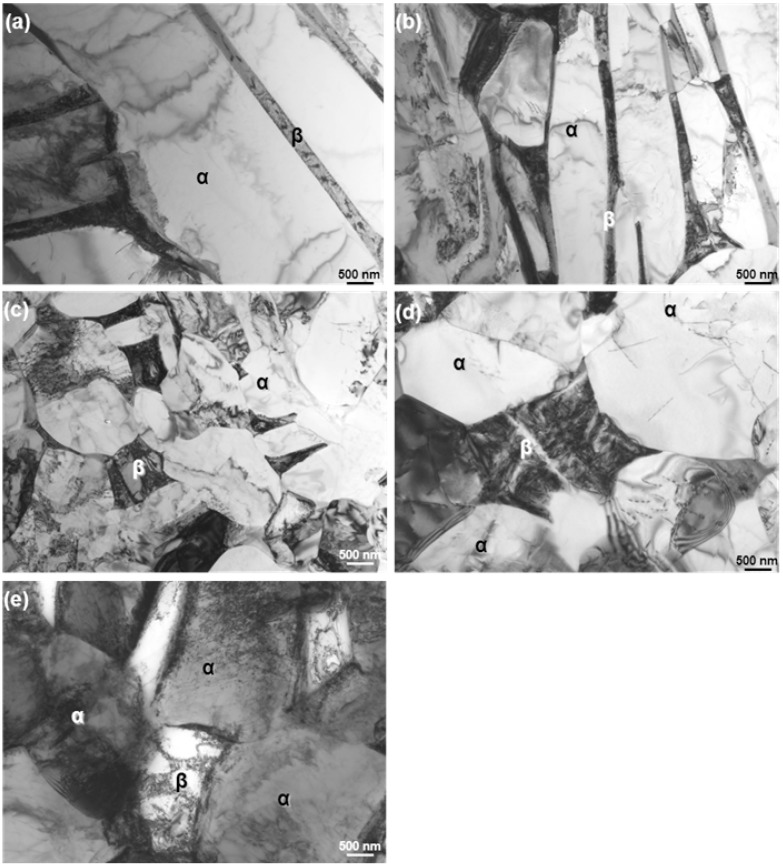
TEM images of (**a–c**) forged Ti-15Zr-4Nb-4Ta alloy A; and (**d**) Ti-15Zr-4Nb-4Ta annealed at 700 °C for 2 h; and (**e**) Ti-6Al-4V annealed alloy at 700 °C for 2 h; (**a**) β-annealed; (**b**) forging ratio of 2 and (**c**) forging ratio of 10.

**Figure 9:**
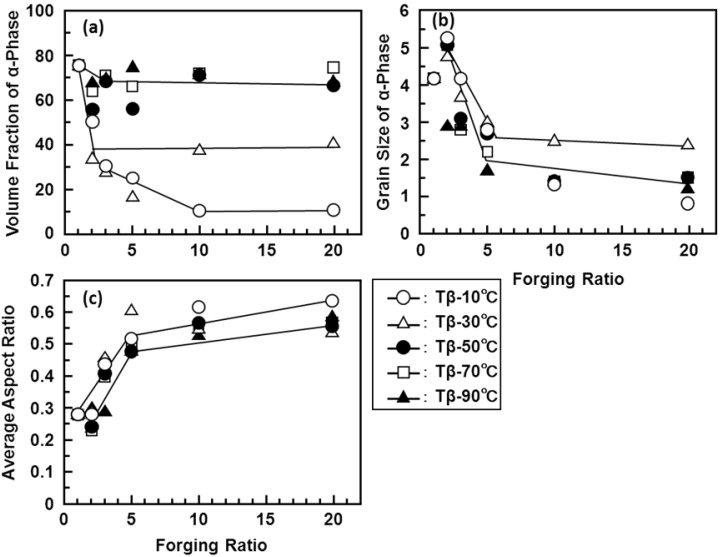
(**a**) Effects of forging ratio and forging temperature on volume fraction of α-phase; (**b**) grain size of α-phase; (**c**) and average aspect ratio of α-phase.

**Figure 10:**
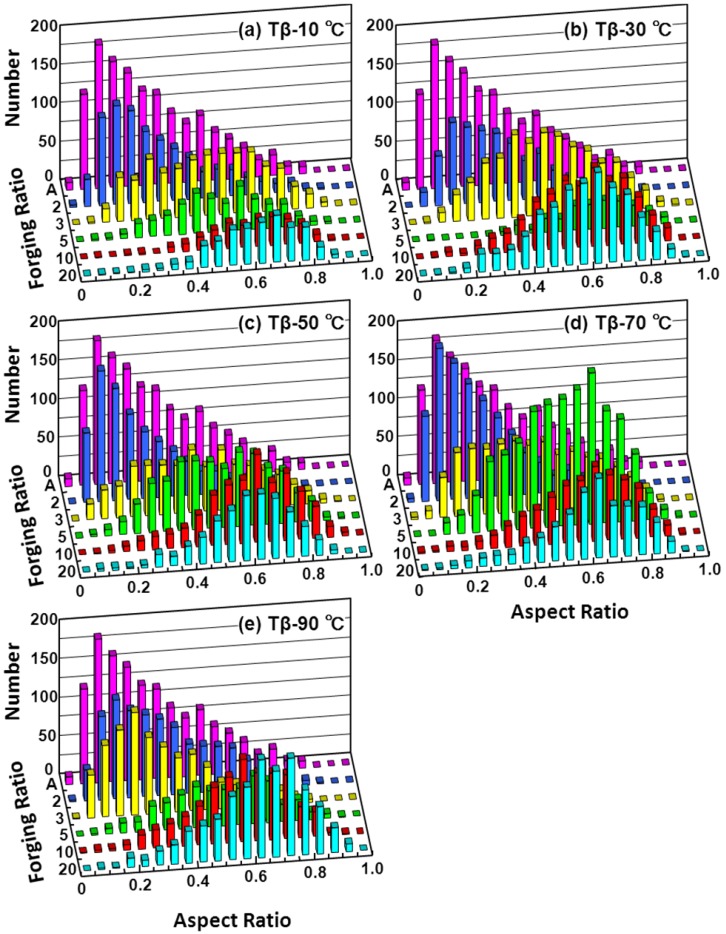
Change in aspect ratio of α-phase as a function of forging ratio and difference (ΔT) between Tβ and heat treatment temperature.

The microstructure of the forged Ti alloy changes with forging ratio (sectional area before forging/sectional area after forging). Figure 7 shows the microstructural change obtained by the α-β forging of the Ti-15Zr-4Nb-4Ta alloy at a starting temperature of 760 °C after β-annealing at 1000 °C for 1 h. The microstructure of the β-annealed Ti-15Zr-4Nb-4Ta alloy consisted of an acicular structure of the α-phase. The acicular structure changed to a granular structure with increasing α-β forging ratio. The microstructure of the Ti-15Zr-4Nb-4Ta alloy forged up to a forging ratio of 10 consisted of an equiaxed fine grain α-phase containing a small amount of β-phase. The rate of transformation to this granular structure tended to increase with increasing heat treatment temperature within a temperature range below Tβ. Figure 8 shows TEM images of the α-β forged Ti-15Zr-4Nb-4Ta alloy. Electron beam diffraction analysis confirmed the β-phase at grain boundaries of the α-phase. In the Ti-15Zr-4Nb-4Ta alloy after β-annealing, an acicular α-phase structure was observed containing a small amount of elongated β-phase at grain boundaries of the acicular α- structure. Otherwise, beyond a forging ratio of 10, a fine granular α-phase structure containing a fine granular β-phase at grain boundaries of an equiaxed α-phase was observed. TEM images of the annealed Ti-15Zr-4Nb-4Ta and Ti-6Al-4V alloys after α-β forging are also compared in Figure 8d,e. A similar α-β structure was seen in both annealed alloys. The effect of forging temperature on the microstructural changes is summarized in Figure 9. The volume fraction of the α-phase increased with increasing temperature difference (ΔT) between β-transus and forging temperature. Its effect was small at forging temperatures below Tβ-50 °C. The grain size of the α-phase markedly decreased with increasing forging ratio up to approximately 5. The aspect ratio of the α-phase markedly increased with increasing forging ratio, and the amount of increase in aspect ratio was small at a forging ratio above 5, as shown in Figure 9c. The effects of forging temperature and forging ratio on the number of aspect ratios of the α-phase are summarized in Figure 10. The aspect ratio after β-annealing was maximum at approximately 0.2. On the other hand, the aspect ratio of approximately 0.75 was maximum at a forging temperature of Tβ-90 °C.

### 3.3. Effects of Forging on Room-Temperature Mechanical Properties of Ti-15Zr-4Nb-4Ta Alloy

**Figure 11:**
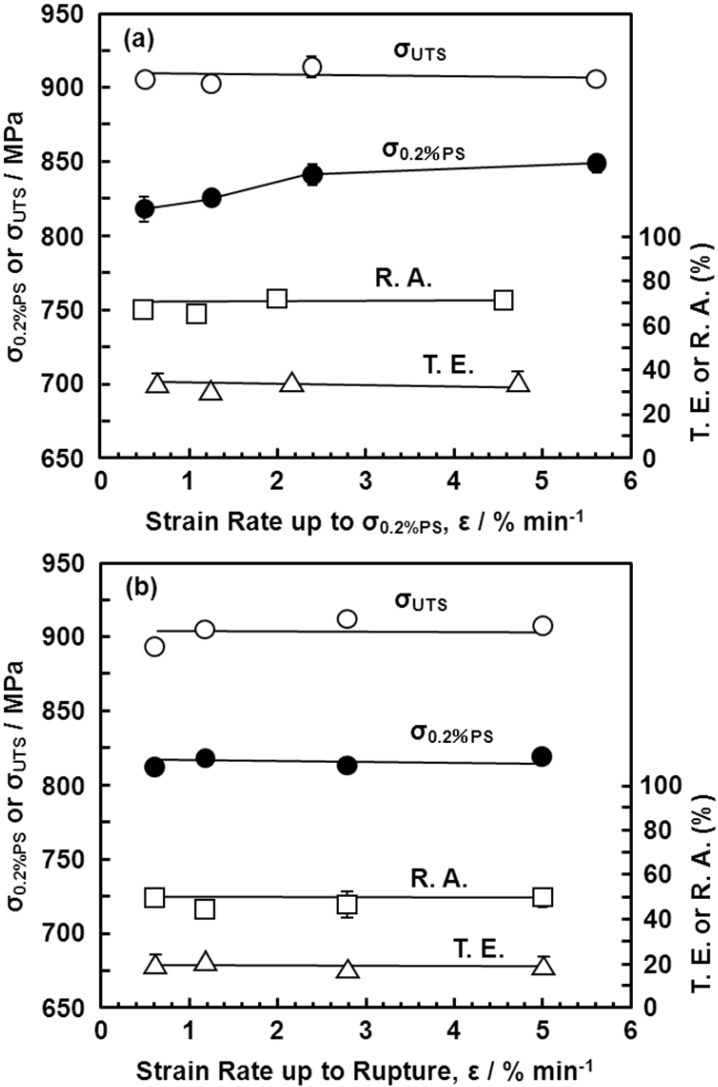
Effects of strain rate (%/mm) on room-temperature mechanical properties (σ_0.2%PS_, σ_UTS_, total elongation (T.E.) and reduction in area (R.A.)). (**a**) Effect of strain rate up to σ_0.2%PS_; (**b**) effect of strain rate from σ_0.2%PS_ up to rupture.

**Figure 12:**
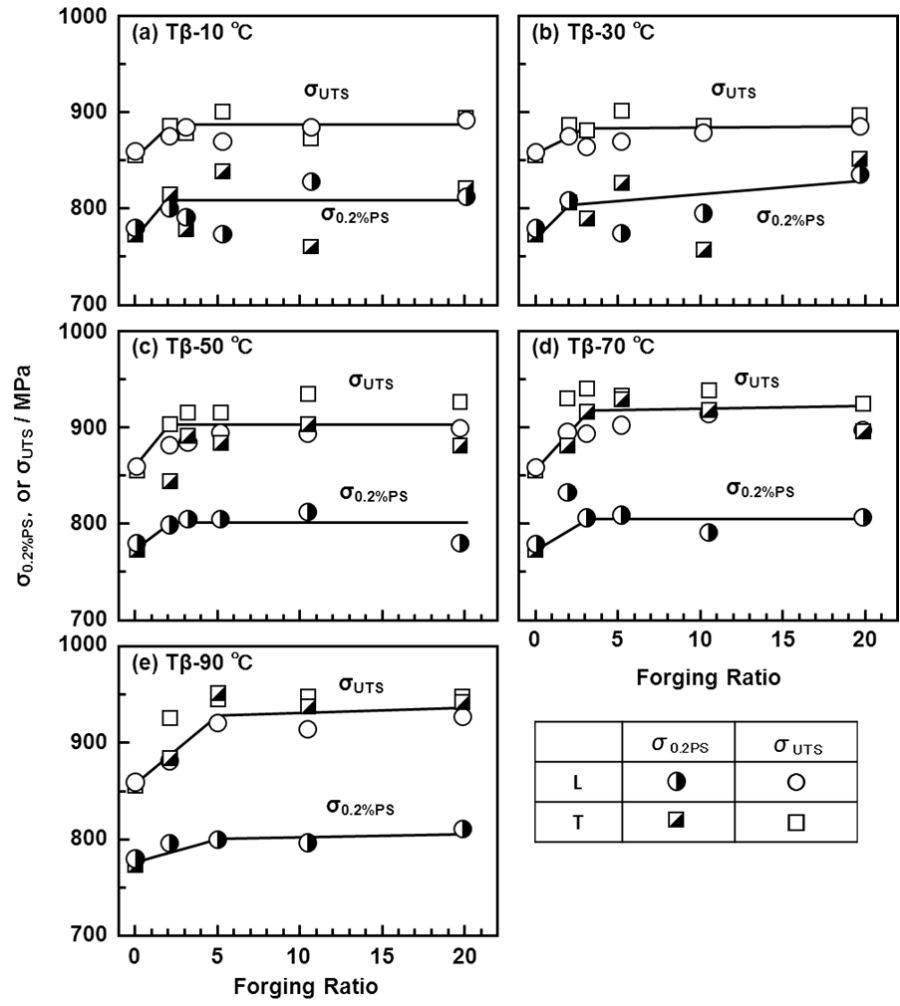
Effects of forging temperature, forging ratio and anisotropy caused by hot forging on σ_0.2%PS_ and σ_UTS_ obtained by room-temperature mechanical test with small specimens (d: 1.5 mm; GL: 9 mm).

**Figure 13:**
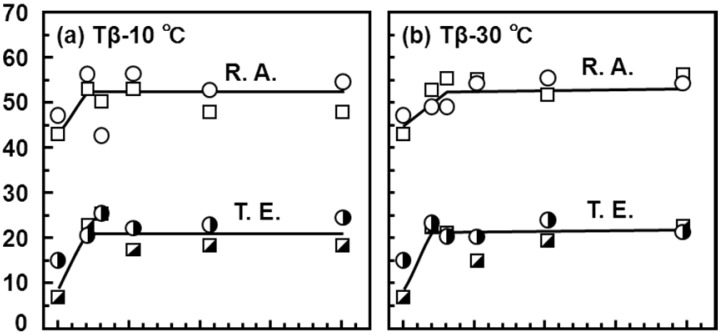

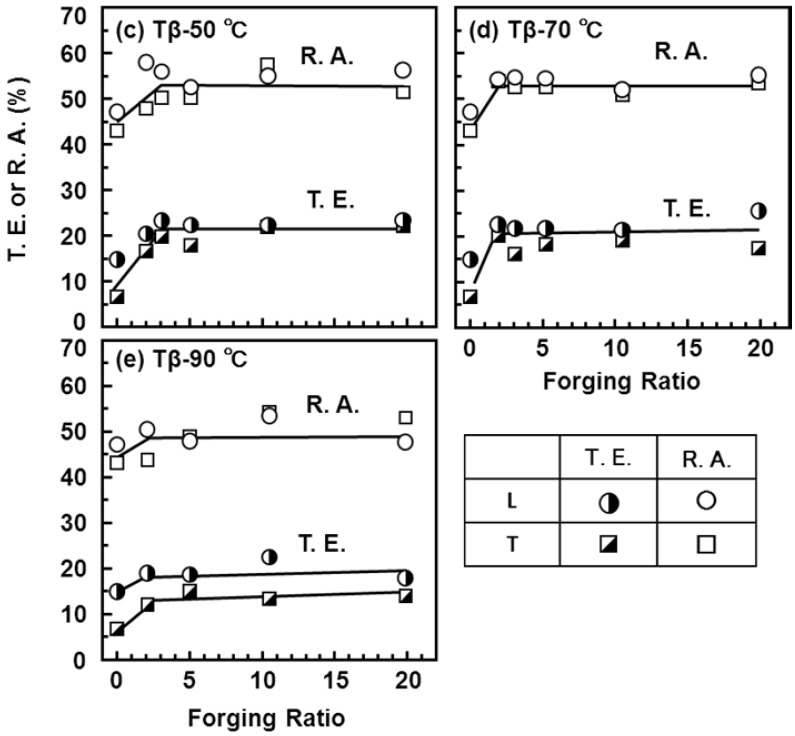
Effects of forging temperature, forging ratio and anisotropy caused by forging on T.E. and R.A. obtained by room-temperature mechanical testing with small specimens (d: 1.5 mm; GL: 9 mm).

**Figure 14:**
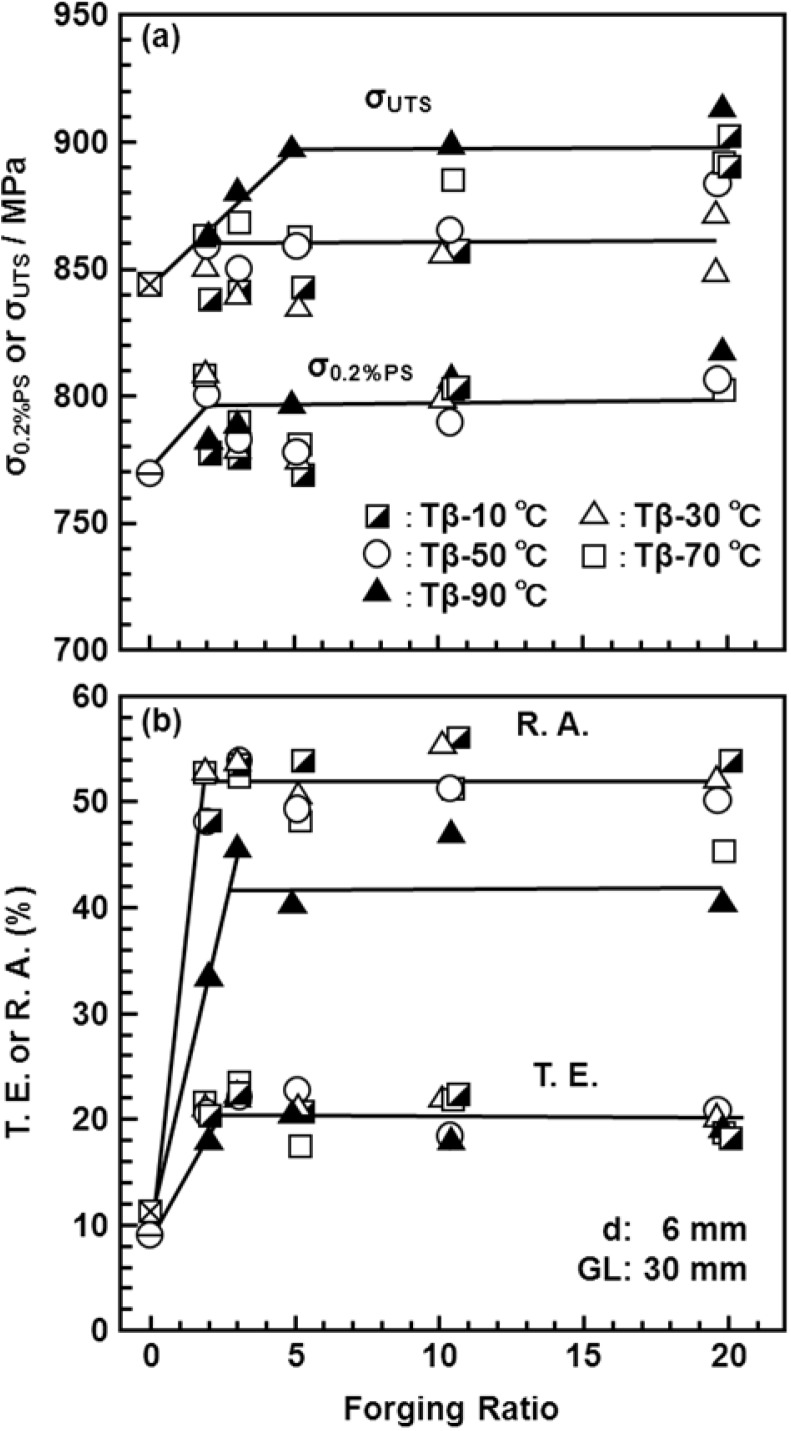
Effects of forging temperature and forging ratio on σ_0.2%PS_, σ_UTS_, T.E. and R.A. obtained by room-temperature mechanical testing with normal-sized test specimen (d: 6 mm; GL: 30 mm).

Figure 11 shows the effects of strain rate on the mechanical properties of the Ti-15Zr-4Nb-4Ta alloy at room temperature. The effects of strain rate on σ_0.2%PS_ and σ_UTS_ were small in the strain rate range of less than 5%/min. Figure 12, Figure 13 show the effects of forging temperature, forging ratio and anisotropy on mechanical properties (σ_0.2%PS_, σ_UTS_, T.E. and R.A.) at room temperature. The effects of forging ratio on σ_0.2%PS_ and σ_UTS_ were small at forging ratios of more than 3 and 5, respectively. When the Ti-15Zr-4Nb-4Ta alloy billet was forged at high temperatures, a high strength was obtained at a lower forging ratio. The effects of forging ratio on T.E. and R.A. were also small at a forging ratio of more than 3. The effect of anisotropy on mechanical properties tended to increase with decreasing forging temperature. For comparison, Figure 14 shows the effects of forging temperature and forging ratio on mechanical properties (σ_0.2%PS_, σ_UTS_, T.E. and R.A.) obtained with a normal specimen at room temperature. A similar tendency was observed in normal-sized specimens. It was considered that Ti alloy with equiaxed fine grains has excellent mechanical properties compared with that with an acicular microstructure. Also, since the mechanical property does not decrease even at a low forging temperature, Ti-15Zr-4Nb-4Ta alloy can be forged for a longer period.

### 3.4. Effect of Forging on Fatigue Strength

**Figure 15:**
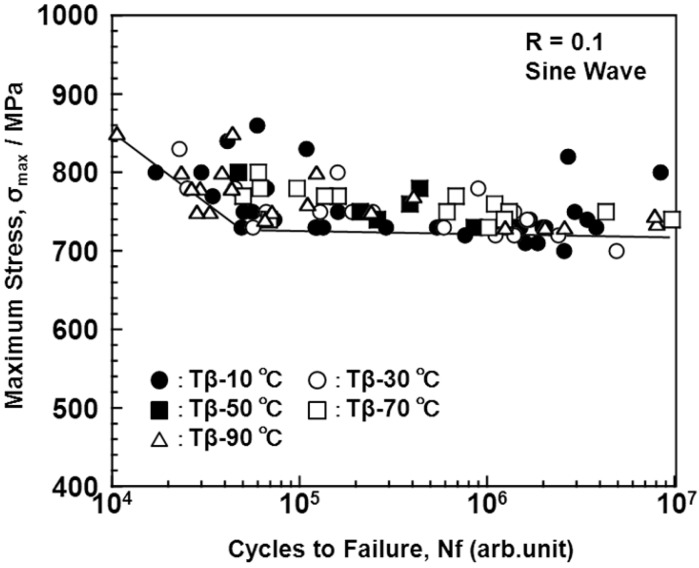
Effect of forging temperature [temperature difference (ΔT) between Tβ and heat treatment temperature] on S-N curves obtained by tension-to-tension fatigue test with sine wave.

Fatigue strength is one of the most important mechanical characteristics of structural biomaterials, because biomaterials are generally used under cyclic loading conditions. Figure 15 shows the S-N curves obtained using different forging temperatures for the Ti-15Zr-4Nb-4Ta alloy hourglass-shaped rods. The fatigue strength at 1 × 10^7^ cycles is approximately 730 MPa. The fatigue strengths of the annealed Ti-6Al-4V hourglass-shaped rods are approximately 685 MPa [24]. It was clear that the relatively high fatigue strength can be achieved by forging in Ti-15Zr-4Nb-4Ta, which consists of the α-β phase.

### 3.5. Manufacturing Equivalency of Ti-15Zr-4Nb-4Ta Alloy

**Figure 16:**
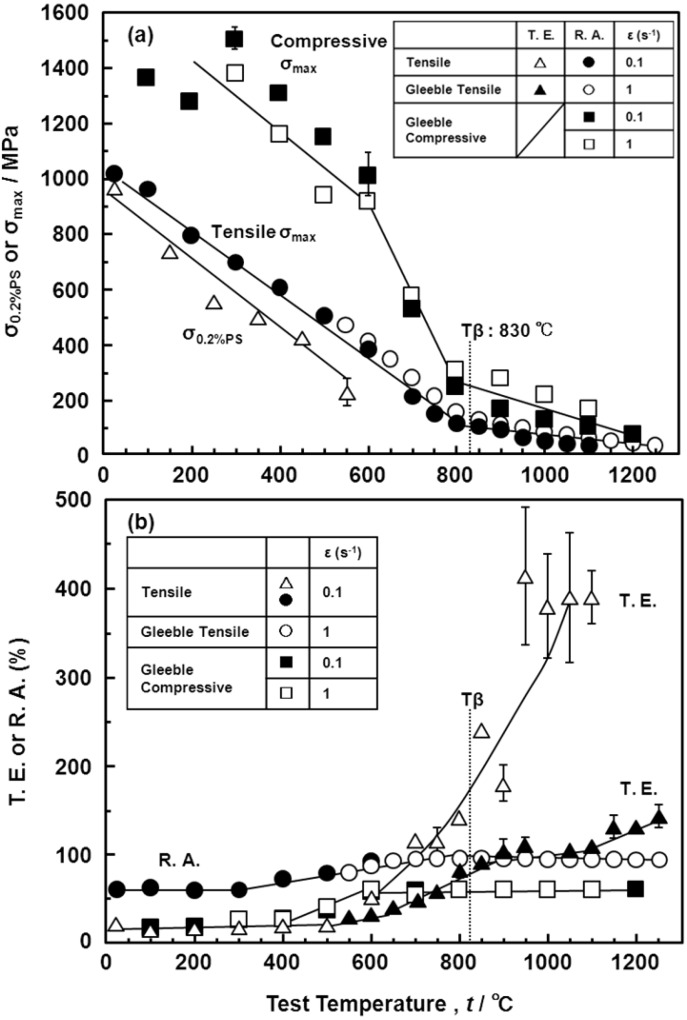
Effects of test temperature on high-temperature mechanical properties (σ_0.2%PS_, σ_max_, T.E. and R.A.).

**Figure 17:**
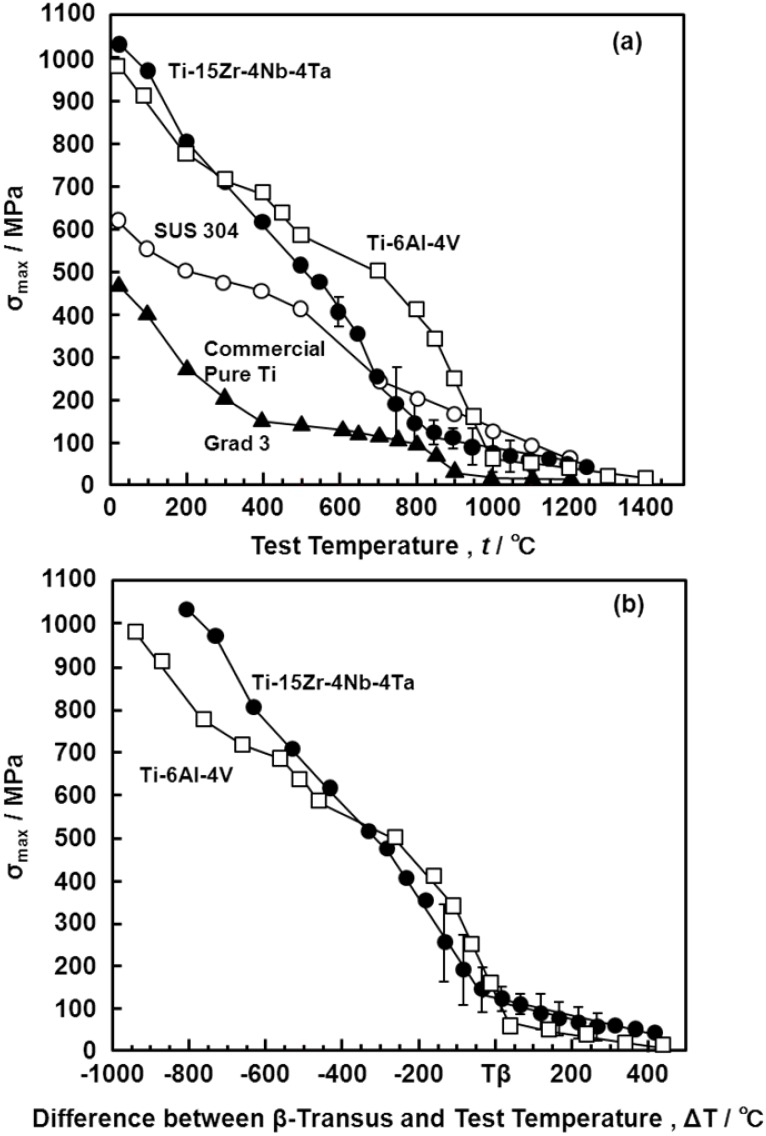
Effect of test temperature on σ_max_ obtained by high-temperature tensile tests. (**a**) Effect of test temperature on σ_max_; (**b**) effect of temperature difference (ΔT) between Tβ and test temperature of tensile test.

**Figure 18:**
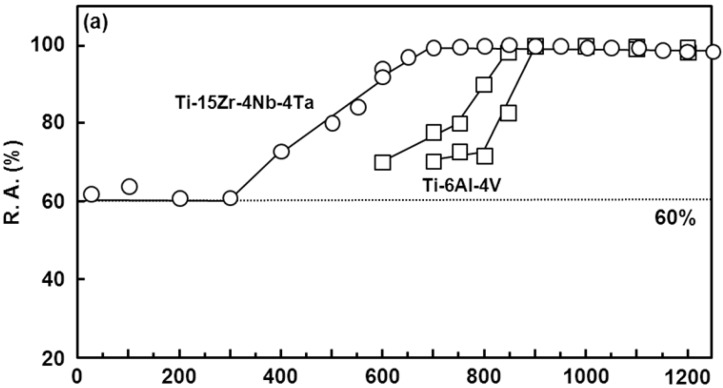

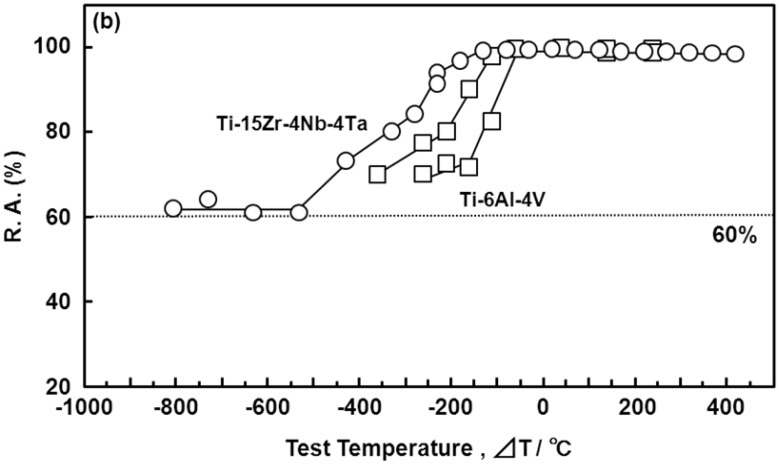
Comparison of R.A. of Ti-15Zr-4Nb-4Ta (B) and Ti-6Al-4V alloys obtained by high-temperature tensile test. (**a**) Effect of test temperature on R.A.; (**b**) effect of temperature difference (ΔT) between Tβ and test temperature of tensile test on R.A.

The hot forgeability of the Ti-15Zr-4Nb-4Ta alloy was excellent, as shown in Figure 1. Since deformation ability is subject to the capacity of the forging machine, hot forgeability was analyzed by a high-temperature tensile test from room temperature up to a high temperature, focusing on changes in σ_max_ and R.A. To compare the hot forgeabilities of the Ti-15Zr-4Nb-4Ta and Ti-6Al-4V alloys, a high-temperature tensile test and three types of Gleeble test (one tensile Gleeble and two compressive Gleeble tests) were conducted. Figure 16 shows the effects of test temperature on the high-temperature mechanical properties (σ_0.2%PS_, σ_max_, T.E. and R.A.) of Ti-15Zr-4Nb-4Ta alloy B. The σ_max_ of the compressive test became significantly higher than that of the tensile test, and the R.A. of the compressive test also became lower than that of the tensile test. The T.E. of the Ti-15Zr-4Nb-4Ta alloy markedly increased with increasing test temperature above 500 °C. σ_max_ markedly increased with decreasing testing temperature below Tβ. On the other hand, R.A. slowly decreased with decreasing testing temperature below Tβ. Figure 17a shows a comparison of the σ_max_ obtained in this study, and those of the Ti-6Al-4V alloy, SUS 304 stainless steel and commercially pure Ti grade 3 reported in the literature [25,26,27,28,29]. Figure 17b shows a comparison of the σ_max_ of the Ti-15Zr-4Nb-4Ta and Ti-6Al-4V alloys with a difference (ΔT) between Tβ and the test temperature. The horizontal axis in Figure 17b shows the difference (ΔT) between Tβ and the test temperature. It was found that the temperature dependences of σ_max_ for the Ti-15Zr-4Nb-4Ta and Ti-6Al-4V alloys are the same and might be a result of the temperature difference (ΔT) from Tβ. The temperature dependences of R.A. for the Ti-15Zr-4Nb-4Ta and Ti-6Al-4V alloys at high temperatures are compared in Figure 18. The temperature dependences of R.A. for both alloys are similar and might be a result of the temperature difference (ΔT) from Tβ. In the Ti-6Al-4V alloy, an R.A. of more than 60% is required as an indication of the hot forgeability of the Ti-6Al-4V alloy [26]. From these results, it was clear that Ti-15Zr-4Nb-4Ta alloy could be manufactured using the same manufacturing process as Ti-6Al-4V alloy taking into account the temperature difference (ΔT) between Tβ and the test temperature. Also, the manufacturing equivalency of Ti-15Zr-4Nb-4Ta alloy to obtain marketing approval of implants was established.

### 3.6. Room-Temperature Mechanical Properties of Hot-rolled Ti-15Zr-4Nb-4Ta Alloy

**Figure 19:**
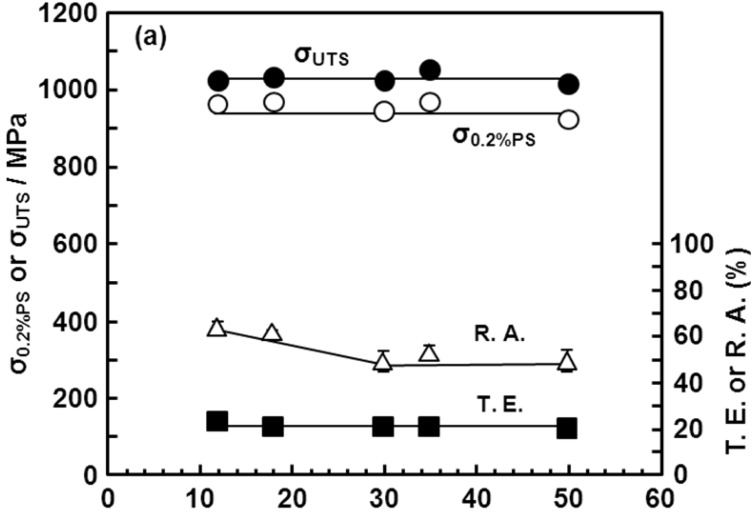

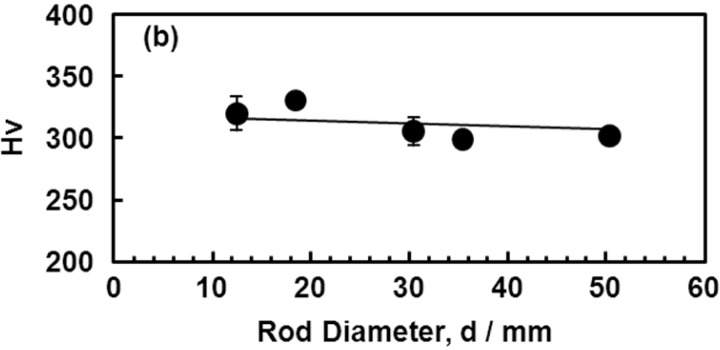
Effects of rolling diameter on mechanical properties (σ_0.2%PS_, σ_UTS_, T.E. and R.A.) and Hv of hot-rolled Ti-15Zr-4Nb-4Ta alloy B.

Figure 19 shows the room-temperature mechanical properties of the continuously hot-rolled Ti-15Zr-4Nb-4Ta alloy rods. High strength, high hardness and excellent ductility for each rod diameter (12 to 50 mm) were obtained in this continuous hot-rolling process. The following formulas are used to express the correlations of the tensile strength of the annealed Ti-15Zr-4Nb-4Ta alloy rods with Vickers hardness and the amount of each alloying element (percent by mass) [30]:

σ_0.2%PS_ (MPa) = −160 + 3.4[Hv], σ_UTS_ (MPa) = −91 + 3.5[Hv] (2)

σ_0.2%PS_ (MPa) = 472 + 9.8[%Zr] − 3.2[%Nb] + 10.7[%Ta] + 335[%O] + 1611[%N] (3)

σ_UTS_ (MPa) = 487 +10.9[%Zr] + 4.9[%Nb] + 2.9[%Ta] + 514[%O] + 1491[%N] (4)

**Figure 20:**
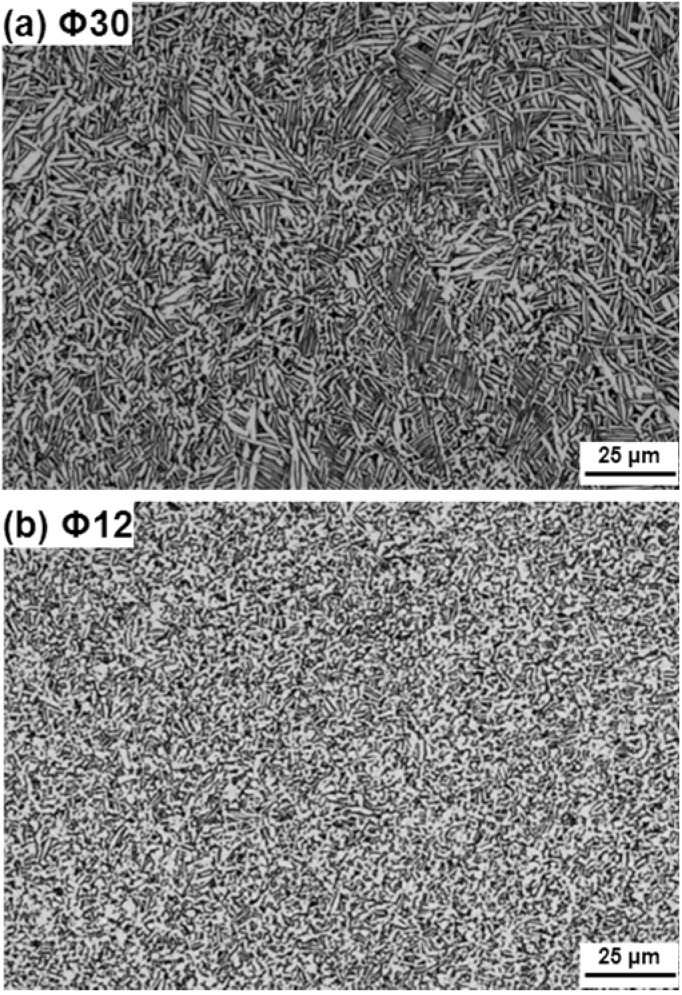
Microscopy images of hot-rolled Ti-15Zr-4Nb-4Ta alloy B. Hot-rolled to diameters of (**a**) 30; and (**b**) 12 mm.

The Ti-15Zr-4Nb-4Ta alloy can be strengthened by adding trace amounts of nitrogen and oxygen. Figure 20 shows the microstructure of the continuously hot-rolled Ti-15Zr-4Nb-4Ta rods. It was considered that equiaxed fine grains produced by the continuous hot rolling exhibited excellent mechanical properties. It was concluded that continuous hot rolling is useful for manufacturing Ti alloys.

Zr, Nb and Ta as alloying elements for Ti alloys are important in attaining superior corrosion resistance and biocompatibility in the long term. The oxides ZrO_2_, Nb_2_O_5_, and Ta_2_O_5_ strengthen passive films and prevent metal ion release, via the addition of Zr, Nb and Ta. However, note that excess addition of Nb and Ta to Ti alloys leads to higher manufacturing cost. The most suitable alloy with the most favourable properties is Ti-15Zr-4Nb-4Ta alloy, which has better room-temperature strength and fatigue properties together as well as biocompatibility. In fact, the biological safety of this alloy has already been proved in cytotoxicity, sensitivity, genetic damage and rabbit implantation tests [24]. This leads to high expectations for the long-term use of this alloy as a viable implant material.

## 4. Conclusions

To prove the manufacturing equivalency of Ti-15Zr-4Nb-4Ta alloy to approved manufacturing processes for Ti-6Al-4V alloy, and to develop low-cost manufacturing processes, the effects of hot-forging and continuous hot-rolling conditions on the microstructure, mechanical properties, hot forgeability and fatigue strength of Ti-15Zr-4Nb-4Ta alloy were investigated. The temperature dependences with a temperature difference (ΔT) from Tβ for the volume fractions of the α- and β-phases for both Ti-15Zr-4Nb-4Ta and Ti-6Al-4V alloys were almost the same. In the α-β-forged Ti-15Zr-4Nb-4Ta alloy, a fine granular α-phase structure containing a fine granular β-phase at grain boundaries of an equiaxed α-phase was observed. The Ti-15Zr-4Nb-4Ta alloy billet forged at Tβ-(30 to 50) °C exhibited high strength and excellent ductility. The effects of forging ratio on mechanical strength and ductility were small at forging ratios of more than 3. The maximum strength (σ_max_) markedly increased with decreasing testing temperature below Tβ. The reduction in area (R.A.) value slowly decreased with decreasing testing temperature below Tβ. The temperature dependences of σ_max_ for Ti-15Zr-4Nb-4Ta and Ti-6Al-4V alloys are the same and might be a result of the temperature difference (ΔT) from Tβ. It was considered that Ti-15Zr-4Nb-4Ta alloy could be manufactured using the same manufacturing process as Ti-6Al-4V alloy, taking into account the temperature difference (ΔT) between Tβ and the heat treatment temperature. Thus, the manufacturing equivalency of Ti-15Zr-4Nb-4Ta alloy to obtain marketing approval was established. Moreover, it was concluded that continuous hot rolling is useful for manufacturing α-β-type Ti alloy.
